# Morpho-physiological and biochemical response of wheat to various treatments of silicon nano-particles under drought stress conditions

**DOI:** 10.1038/s41598-023-29784-6

**Published:** 2023-02-15

**Authors:** Muhammad Aown Sammar Raza, Bilal Zulfiqar, Rashid Iqbal, Muhammad Noor Muzamil, Muhammad Usman Aslam, Faqeer Muhammad, Jawad Amin, Hafiz Muhammad Usman Aslam, Muhammad Arif Ibrahim, Muhammad Uzair, Muhammad Habib-ur-Rahman

**Affiliations:** 1 Department of Agronomy, Faculty of Agriculture and Environment, The Islamia University of Bahawalpur, Bahawalpur, 63100 Pakistan; 2 Department of Agronomy, MNS-University of Agriculture, Multan, 60000 Pakistan; 3 Institute of Plant Protection (IPP), MNS-University of Agriculture, Multan, Pakistan; 4 National Institute for Genomics and Advanced Biotechnology (NIGAB), National Agricultural Research Centre (NARC), Park Road, Islamabad, 45500 Pakistan; 5 Crop Science Group, Institute of Crop Science and Resource Conservation (INRES), University of Bonn, Bonn, Germany

**Keywords:** Plant sciences, Environmental sciences

## Abstract

Silicon nanoparticles (Si-NPs) have shown their potential for use in farming under water-deficient conditions. Thus, the experiment was accomplished to explore the impacts of seed priming of Si-NPs on wheat (*Triticum aestivum* L.) growth and yield under different drought levels. The plants were grown in pots under natural ecological environmental conditions and were harvested on 25th of April, 2020. The results revealed that seed priming of Si-NPs (0, 300, 600, and 900 mg/L) suggestively improved, the spike length, grains per spike, 1000 grains weight, plant height, grain yield, and biological yield by 12–42%, 14–54%, 5–49%, 5–41%, 17–62%, and 21–64%, respectively, relative to the control. The Si-NPs improved the leaf gas trade ascribes and chlorophyll *a* and *b* concentrations, though decreased the oxidative pressure in leaves which was demonstrated by the diminished electrolyte leakage and upgrade in superoxide dismutase and peroxidase activities in leaf under Si-NPs remedies over the control. The outcomes proposed that Si-NPs could improve the yield of wheat under a dry spell. In this manner, the utilization of Si-NPs by seed priming technique is a practical methodology for controlling the drought stress in wheat. These findings will provide the basis for future research and helpful to improve the food security under drought and heat related challenges.

## Introduction

Drought is striking ecological stress among other abiotic stresses, which adversely influence crop growth and yield of the crops around the world^1^. Productivity of crops is diminishing because of restricted stock of water^2^, less intake of nutrients, and poor photosynthesis^3^. Even though drought effects wheat performance at all growth stages, it is more rudimentary during the blooming and grain-filling stages (terminal dry season), which results in considerable yield loss. The leading explanations behind these losses are the diminished rate of net photosynthesis due to metabolic limitations, oxidative impairment to chloroplasts and stomatal closing, and abandoned grain set and improvement^4^. Nonetheless, drought stress animates the creation of ethylene^5,6^ which delay and slow down the root growth and prolongation^2,7^. All things considered, plants have established different versatile mechanisms to acclimatize the water shortage by prompting a succession of physiological and biochemical reactions^8^. Among these components, antioxidants play a vital role in reducing oxidative damage caused by drought^9^.

Drought impact in Pakistan is more significant because of its massive reliance on agribusiness. Besides this, Pakistan become more vulnerable to drought due to a lack of stress awareness and control practices^10^, and high risk in the future due to recent changes in climate^11,12^. Wheat is a major rabi season crop which is contributing 8.7% of the worth expansion in agribusiness and 1.7% to Pakistan’s GDP. The territory under wheat growth in Pakistan during 2019–2020 was 8825 thousand hectares with a growth of 1.7% when contrasted with the space of a year ago which was 8678 thousand hectares. Moreover, the estimated production of wheat was 24.946 million tons in 2019 which was 2.5% higher than the previous year^13,14^.

Over half of the world’s wheat fields are influenced by periodic drought stress^15^. The rapid climate change and global warming have the worst impact on the crops in the future because it enhances the frequency and intensity of drought due to less rainfall and high temperature on earth^16^. Accordingly, for enhancing the yield and preserving the wheat from such stresses need to develop more understanding and approaches to overcome these issues^17^.

In the last decade, nanoparticles play an imperative role in agriculture due to their unique properties, such as greater absorption ability, higher surface area, efficient delivery method for a specific spot, and such technology used in many ways like additives, plant growth enhancers, nano-fertilizers, and plant protection agents^18,19^. Sedghi et al.^20^ stated that the utilization of silicon nanoparticles is assuming a significant part in the plant resistance against the shortage of water over expanding amino acids, chlorophyll *b* contents, lipids, and proteins. Among other NPs, the Si-NPs are helpful in elevation the plant growth and photosynthesis under an intensive atmosphere^21,22^.

Nanoparticles application expanded the growth, chlorophyll substances, and gas trade qualities in the wheat grain and diminished the water deficiency^23^. Between various nanoparticles, silica nanoparticles play a magnificent role by increasing the photosynthesis that enhances the yield of *Cucurbita pepo* L.^24^ and hawthorn seedlings^25^ under stress conditions. Many types of research indicated that plant growth, antioxidant activities, and nutrient uptake process under drought stress was faster with the application of nanoparticles because such particles enhance the resistance of plant^21,26–28^. Si-NPs improve photosynthesis parameters of certain plants during dry season and has a greater impact in agriculture as compared to other fertilizers^29–31^. The exogenous application of NPs provoke root endodermal silicification, and antioxidant system activity and manage the cell water stability^32^. Nano-silicon application has improved production and growth and enhanced plant water status with extraordinary changes in the ultrastructure of leaf organelles, initiation of plant protection and defense system and searching of unambiguous particles^33^. Besides, SiO_2_ NPs have been described as a plant growth inducer, which raises root endodermal silicification, upgrades antioxidant system under pressure, and improve resilience to abiotic stresses and the cell water balance^32^. The connection between SiO_2_ and plant cell wall has been reported in numerous monocots^34^.

Nano-particles can be applied in many ways under the biotic and abiotic conditions in the field of agriculture such as by seed priming, soil, and foliar application, but seed priming is the most efficient method^23,35–37^. Seed priming is a fruitful strategy in many ways, for example by eliminating the worst effect of abiotic stresses, stimulating the valuable physiological changes, helping at the initial stage of germination, enhancing seed germination rate, minimizing the seedling emergence time, and most importantly it is a cost-effective technique for farmers^38–43^. There is no literature regarding effect of Si-NPs on wheat morpho-physiological and biochemical attributes under drought at different growth stages. So, the objective of this study is to mitigate the catastrophic effects of drought on wheat under drought conditions by the application of silicon nanoparticles.

## Materials and methods

### Experimental design

The research work was carried out in the wirehouse, department of agronomy, The Islamia University of Bahawalpur, Pakistan (Latitude: 29° 23′ 60.00 N, Longitude: 71° 40′ 59.99 E). The experimental layout was a randomized complete block design with four replications of each treatment of silicon nanoparticles (0, 300, 600, and 900 mg/L). All pots were irrigated equally till complete emergence, after that water deficient condition was executed at critical growth stages, such as tillering, flowering, and grain filling stages, while complete irrigation was considered as control.

### Silicon nanoparticles seed priming and drought imposition

The wheat seeds (Glaxy-2013) were sterilized with a solution of sodium hypochlorite (2.6% active chlorine) for 120 s and washed thrice with distilled water. Then the measured amount of silicon nanoparticles, Sigma-Aldrich supplied spherical Si-NPs with a particle size in the 40–80 nm range^43^, was mixed with deionized water and ultra-sonication of each Si-NPs treatment (0, 300, 600, and 900 mg/L) for thirty minutes for proper dispersion was done. The wheat seeds were then soaked in NPs solutions for 20 h in dark under room temperature. For control treatment seeds were soaked in deionized water. Finally, dried the primed seed of the wheat crop and stored at 4 °C for further experiment.

### Crop husbandry

**Figure 1:**
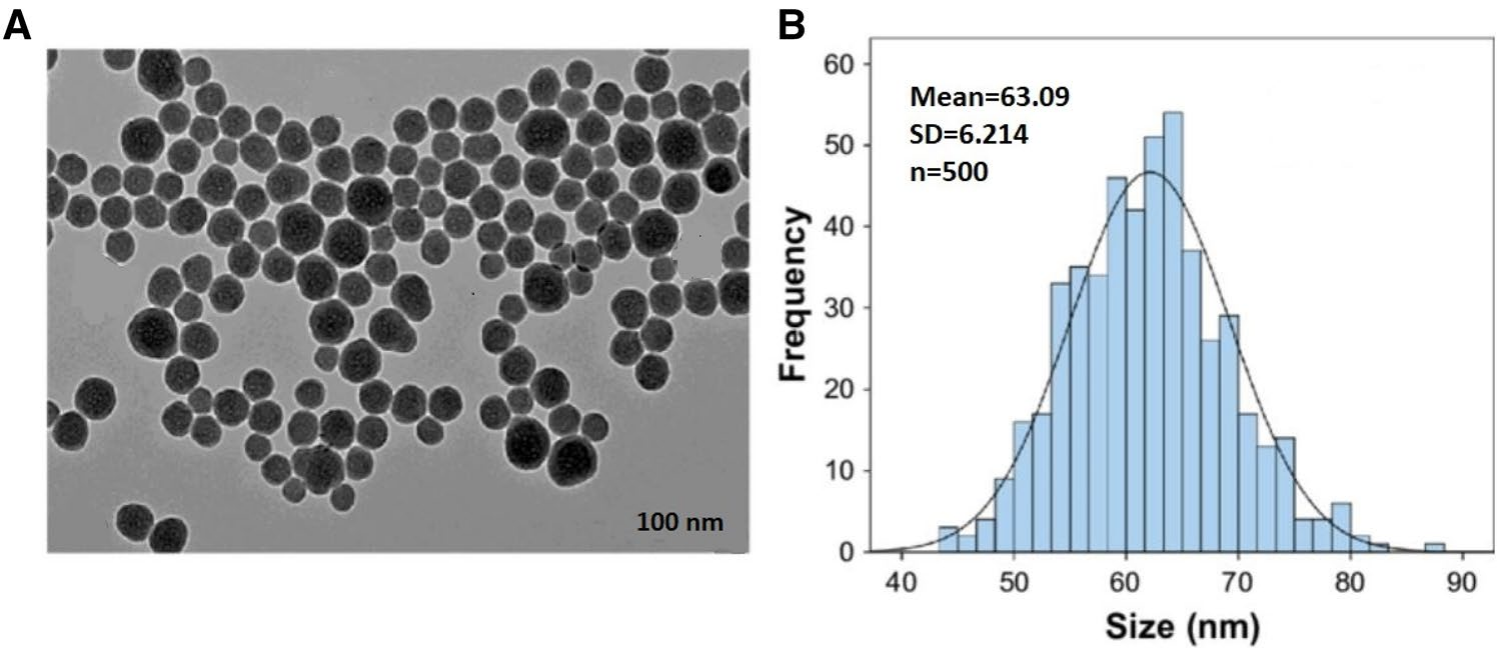
Si-NP characterization. TEM scans indicated well-dispersed spherical particles (**A**). (**B**) Normal distribution graph with 63.09 ± 6.21 nm mean diameter.

In the present study, Si-NPs was purchased from Sigma-Aldrich. In addition to having a purity of > 99%, Si-NPs had a specific surface area of > 80 m^2^g^−1^ and 28.08 molecular weight. Scanning Electron Microscope was used to measure the NPs’ sizes (Fig. 1). The mean size of the main particles was 63.09 ± 6.2 nm.

The seeds of the indigenous wheat cultivar (Galaxy-2013) were obtained from Regional Agricultural Research Institute (RARI) Bahawalpur, as it is lodging and heat resistant. Wheat seeds were primed with Si-NPs and planted in plastic pots (26 × 29 cm) filled with clayey loam soil on 9th November 2019 and a physio-chemical investigation of the soil was done before planting (Table 1). The wirehouse was covered with a transparent plastic sheet to preserve the plants from rain, when mandatory. The plant population was maintained as 4 plants per pot after 20 days of sowing and irrigated equally till complete emergence of seedlings. After that, 30% water holding capacity (WHC) was maintained in pots under drought at three observed stages and 80% WHC was considered as control.

**Table 1:** Physical and chemical properties of the experimental soil.

Parameters	Soil profile
Sand	56.5%
Silt	32.5%
Clay	11%
Texture class	Sandy loam soil
Ph	7.23
Electric conductivity (dSm^−1^)	2.55
Ammoniac N (mg g^−1^)	1.58
Organic matter (%)	0.92
Available phosphorus (ppm)	6.75
Available potassium (ppm)	112

### Growth parameters and measurement

Wheat growth, yield, and yield attributes such as spike length (cm), number of grains per spike, 1000-grain weight (g), plant height (cm), organic yield per plant (g), grain yield per plant (g), and harvest index were calculated following the standard procedures and protocols. For yield, wheat plants were manually harvested after the completion of the life cycle. Similarly, for measuring the plant height, four plants were arbitrarily selected from each pot and measured the height from soil surface to spikelet with the help of a ruler having read in millimeters and centimeters. For measuring the spike length and grains per spike, used a centimeter scale measuring the length from base to terminal spikelet from 10 plants randomly and counted the grains from randomly selected ten clusters of seeds. Besides, to quantify 1000-grain weight, the digital balance was used due to its accuracy of 0.01 g.

### Relative water content (RWC)

For measuring the RWC, 5 mature fresh flag leaves were taken randomly from each treatment and immediately placed in polyethylene bags and taken to the laboratory. Where, firstly, get the fresh weight of taken samples quickly before any dehydration. Then, the sample was placed in distilled water for getting a turgid weight. After 3 h, the sample was taken out from distilled water and measured its turgid weight. After that sample was placed in an oven for 2 days at 65 °C and obtained its dry weight. Finally, the relative water content of each treatment was measured by utilizing the below-given formula^44^:

$$ {\text{RWC \% }} = \frac{{{\text{FW}} - {\text{DW}}}}{{{\text{TW }} - {\text{DW}}}} \times 100 $$

where RWC, FW, DW, and TW are relative water contents, fresh weight, dry weight, and turgid weight of the leaf’s samples.

### Superoxide dismutase and peroxidase in wheat leaves

The superoxide dismutase and peroxidase were measured with the help of a spectrophotometer. Fresh leaf material 0.5 g from each treatment was homogenized with 50 mM phosphate buffer with 7.5 pH and 1 mM dithiothreitol (DTT) under chilled conditions after washing and drying the sample with distal water^45^. SOD activities were measured by using the procedure published by Cakmak^46,47^, whereas POD was determined by the method of Zhang and Kirkham^48^.

### Chlorophyll fluorescence

The photosynthetic pigments chlorophyll (Chl *a*, and Chl *b*) were extracted in 80% (v/v) acetone and measured spectrophotometrically (Spectronic 601, Milton Roy Company, Rochester, NY, USA) according to Metzner et al.^49^. Leaf Chl fluorescence was calculated utilizing a pulse amplitude modulation portable fluorometer (Handy PEA. Hansatech. Norfolk, UK). The leaves were dark and adapted in a leaf clip for 30 min. For all treatments, 30 measurements (three replicates of 10 leaves from various plants) were recorded. The data acquired were utilized to figure out the maximum efficiency of PS II (FV/FM) and performance index on an absorption basis (Plans)^50^ according to the equations reviewed by Stirbet^51^.

### Statistical analysis

STATISTIX software (version 8.1) was run on present data to determine the analysis of variance (ANOVA) and 5% LSD was used as the probability for the mean comparison of data^52^. Data was then analyzed for principal component analysis (PCA) by R software (R Core Team, 2019) to check the association among different studied morpho-physiological attributes.

### Plant guidelines

All the plant experiments were in compliance with relevant institutional, national, and international guidelines and legislations.

## Results

The result of the combined analysis of variance showed that Silicon nanoparticles concentration, drought intervals, and control irrigation regimes significantly affected all measured traits. There was a critical two-way connection between NPs concentration and drought on enzyme action, SOD, and POD. Additionally, the interaction among nanoparticles concentration, drought, and irrigation regimes was significant for spike length, the number of grains per spike, 1000-grain weight, plant height, grain yield, and biological yield. Besides, there was significant correlation between NPs concentration and control, besides NPs concentration and drought interaction on photosynthesis rate, transpiration rate, stomatal conductance, and RWC content. Moreover, the percentage of nitrogen, phosphorus, and potassium was affected by the triple association between the drought, control, and nanoparticles treatment.

### Effect of silicon nanoparticles and drought on plant height

Silicon nanoparticles played a major share in plant growth under drought conditions at critical periods of wheat growth, when analyzed with control treatments. Plant height was reduced by 38.25% in DTS, 9.07% in DFS, and 6.77% in DGFS compared to control watering. When compared to the control (0 mg/L NPs), applications of 300, 600, and 900 mg/L of silicon nanoparticles mitigate the negative effects of drought and increase plant height by 2.5%, 3.2%, and 6.9%, respectively. The results showed that seed priming with 900 mg/L Si-NPs was the most effective, whereas the 0 mg/L Si-NPs treatment resulted in the shortest plant heights.

### Effect of silicon nanoparticles and drought on yield attributes

The yield attributes were significantly influenced by the application of Si-NPs under water-deficient conditions shown in Table 2. Drought stress decreased the spike length (27.81, 21.18, and 22.82%), the number of grains per spike (26.19, 25.43, and 22.9%), 1000 grain weight (48.22, 42.37, and 40.31%), biological yield (10.54, 10.17 and 6.04%), grain yield (83.59, 79.56 and 66.22%) and HI (67.7, 64.07 and 57.3%) in DGFS, DFS, and DTS, respectively, with compare to control treatment. Silicon nanoparticle applications alleviated the adverse effects of water deficiency under control and stress environments. The use of silicon nanoparticles enhanced the entire above-mentioned yield attributes fundamentally, contrasted with control treatment. Moreover, compared with the control application, most higher grain production (42.12%) was recorded with priming of 900 mg/L silicon nanoparticle.

**Table 2:** Effects of Si-NPs on different parameters of wheat under drought stress.

Treatments	Si-NPs application	PH	SL	NGPS	GW	GY	BY	HI
CK	SiNP0 = 0 mg/L	66.7 a	6.43 bc	33.2 a	32.3 a	22.1 b	60.0 cdef	37.0 a
	SiNP1 = 300 mg/L	65.9 a	6.97 a	33.0 a	34.0 a	24.5 ab	65.0 abc	37.8 a
	SiNP2 = 600 mg/L	67.0 a	6.73ab	36.4 a	35.1 a	26.5a	68.0 a	38.9 a
	SiNP3 = 900 mg/L	70.2 a	7.13 a	36.3 a	34.4 a	25.3 a	67.0 ab	37.8 a
DTS	SiNP0 = 0 mg/L	44.9 def	4.90 h	22.3 cd	18.5 d	10.5 gh	55.0 fg	19.1 de
	SiNP1 = 300 mg/L	47.8 cde	5.50 efg	26.2 bc	23.6 c	12.5 fgh	58.0 defg	21.5 cde
	SiNP2 = 600 mg/L	47.8 cde	5.87 de	27.3 b	25.4 bc	16.4 cd	62.0 bcde	26.5 bc
	SiNP3 = 900 mg/L	54.5 b	5.93 de	27.8 b	27.9 b	15.5 cdef	61.0 cde	25.4 bc
DFS	SiNP0 = 0 mg/L	43.3 ef	5.07 gh	25.2 bcd	19.9 d	11.0 gh	57.33 efg	19.3 de
	SiNP1 = 300 mg/L	43.9 ef	5.80 de	26.3 bc	24.2 c	13.5 defg	60.0 cdef	22.7 bcde
	SiNP2 = 600 mg/L	48.1 cde	5.57 ef	26.4 bc	24.9 c	17.7 c	63.0 abcd	28.1 b
	SiNP3 = 900 mg/L	55.3 b	6.07 cd	27.8 b	27. 9b	17.0 c	65.0 abc	26.4 bc
DGFS	SiNP0 = 0 mg/L	41.5 f	5.13 fgh	21.5 d	18.0 d	9.5h	53.0g	17.9 e
	SiNP1 = 300 mg/L	45.0 def	5.07 gh	24.6 bcd	23.5 c	13.0efg	60.0cdef	21. cde
	SiNP2 = 600 mg/L	50.7 bcd	5.2 3fgh	27.7 b	23.9 c	15.0cdef	62.0bcde	24.2 bcd
	SiNP3 = 900 mg/L	53.1 bc	5.90d e	28.7 b	26.3 bc	16.0cde	60.0cdef	26.7 bc
LSD (*P* ≤ 0.05)		5.0977	0.4666	4.2912	2.9595	299.18	1267.3	5.6913

Plant height (PH, cm), spike length (SL, cm), number of grains per spike (NGPS), 1000-Grain Weight (GW, g), Grain Yield per Plant (GY, g), Biological Yield per Plant (BY, g), Harvest Index (HI) affected by Si-NPs application under drought stress conditions at different growth stages of wheat (*CK* Control, *DTS* Drought at tillering stage, *DFS* drought at flowering stage, and *DGFS* Drought at grain filling stage).

### Effect of silicon nanoparticles and drought on chlorophyll contents

**Figure 2:**
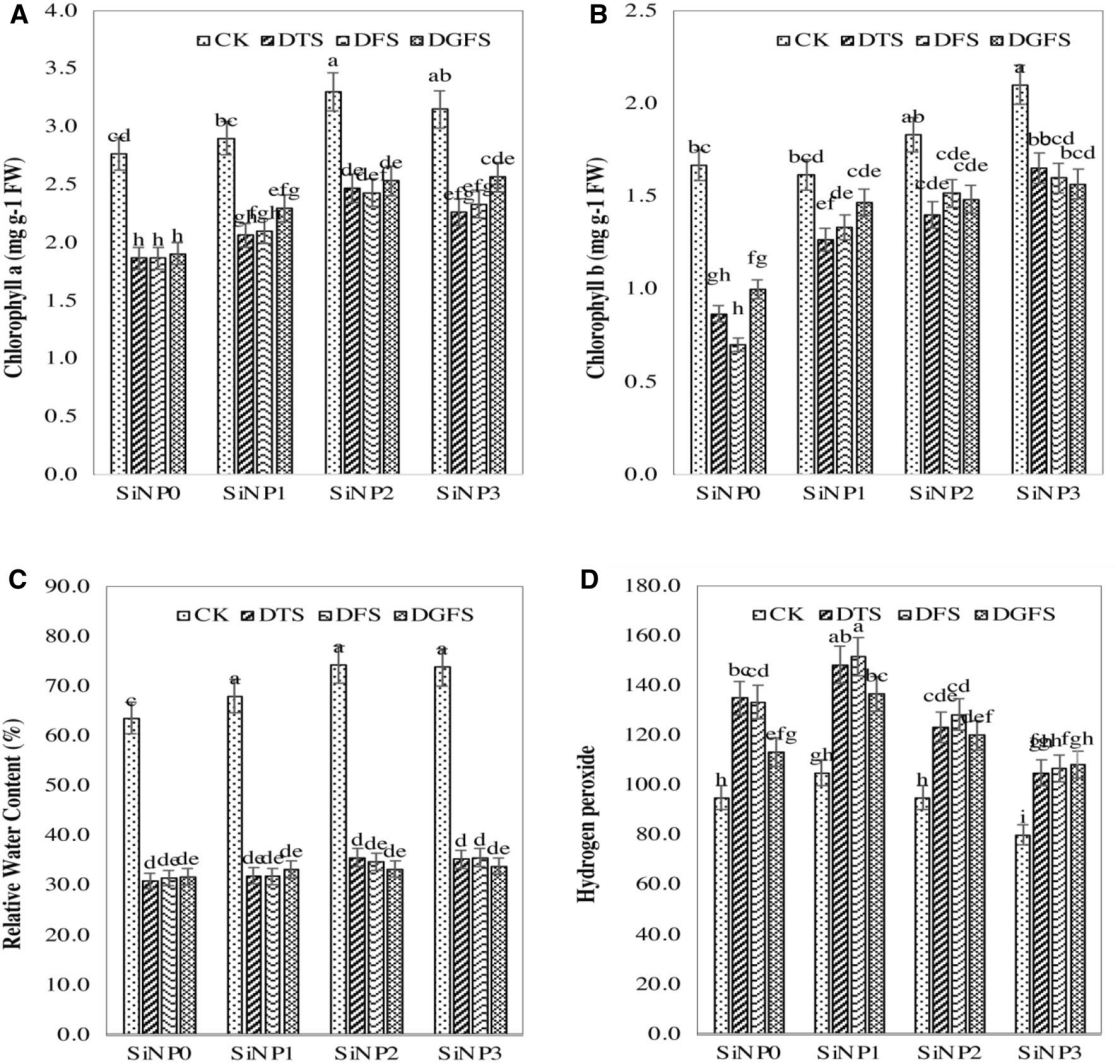
Chlorophyll contents measurements. (**A**,**B**) Chlorophyll a and b contents (**C**,**D**) Relative water contents and H_2_O_2_affected by Si-NPs (SINP0 = 0, SINP1 = 300, SINP2 = 600, and SINP3 = 900 mg/L) application under drought stress at critical growth stages (CK = control, DTS = drought at tillering stage, DFS = drought at flowering stage, DGFS = drought at grain filling stage) of wheat.

Seed preparation with silicon nanoparticles beneficially influenced the chlorophyll contents of wheat leaves grown under drought stress (Fig. 2A,B). Compared to well-watered plants (control), drought causes a significant reduction in chlorophyll a (30.2, 38.7, and 39.8%) and b (30.8. 40.1 and 39.2%) contents in DGFS, DFS, and DTS, respectively. Chlorophyll a and b substance was expanded significantly when nanoparticles were applied. However, the seeds were primed with a 900 mg/L solution of Si-NPs showing the highest amount of chlorophyll contents.

### Effect of silicon nanoparticles and drought on RWC and H_2_O_2_

Drought stress caused a significant reduction in RWC (Fig. 2C) as the crop goes to maturity. Usage of NPs, particularly 900 mg/L, increased RWC compared with the control (NPs = 0 mg/L). Application of 900 mg/L Si-NPs to plants under normal conditions (without stress) and the treatment without Si-NPs at tillering stage in wheat plants under water-deficient conditions caused the highest RWC (74.3%) and the lowest RWC (31.8%), respectively. On the other hand, drought positively reduced the mean of RWC compared with normal irrigation, while the utilization of NPs in plants under drought stress beneficially enhanced the mean of RWC of leaves (4.6–13.5%) compared with the control. H_2_O_2_ is also affected as the crop goes to later stages (Fig. 2D).

### Effect of silicon nanoparticles and drought on gas exchange attributes

**Figure 3:**
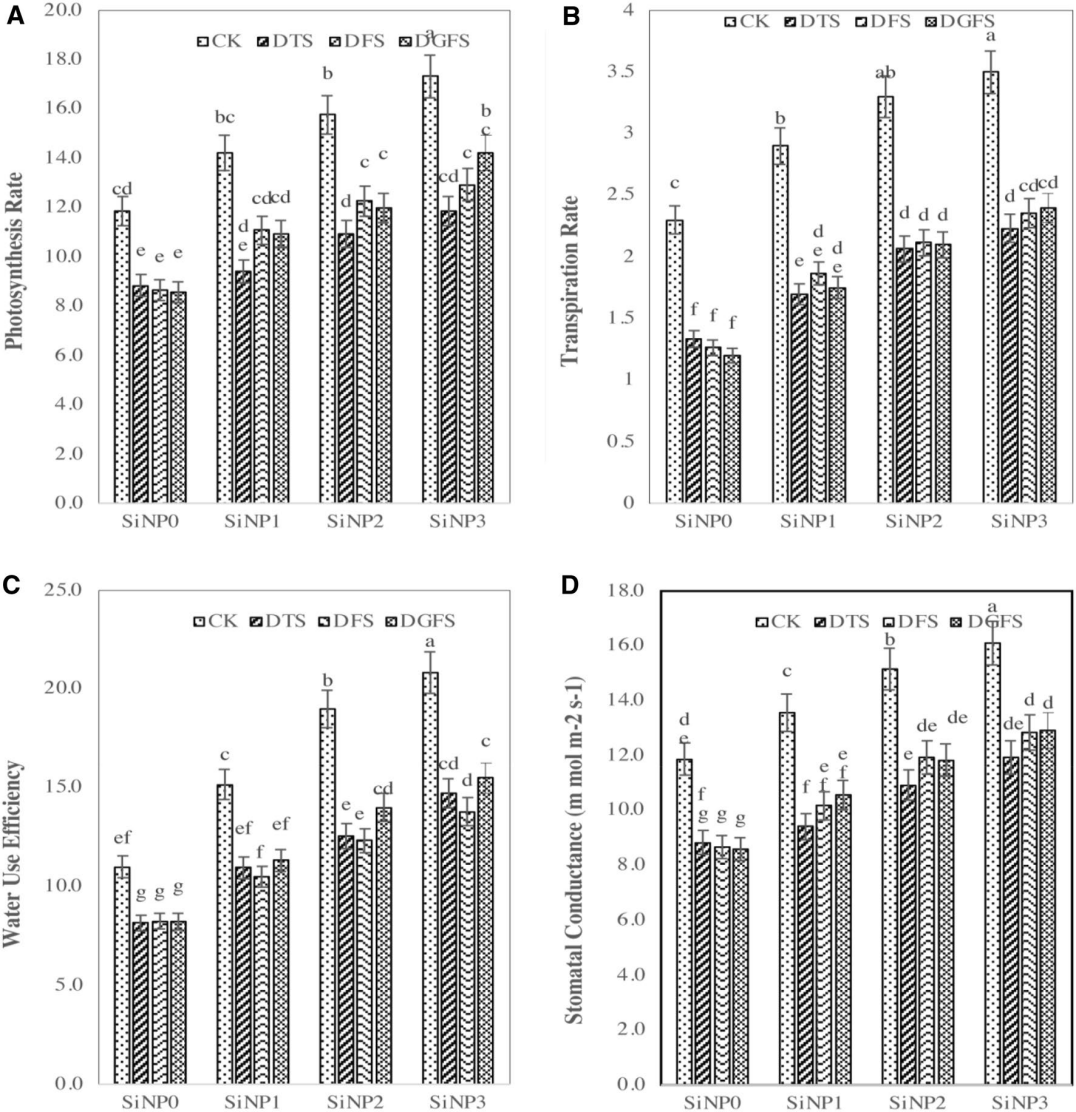
Photosynthesis rate (Pr, **A**), transpiration rate (Tr, **B**), Water use efficiency (WUE, **C**), and stomatal conductance (SC, **D**) contents affected by Si-NPs application (0, 300, 600, and 900 mg/L) under drought stress at critical growth stages (*CK* Control, *DTS* Drought at tillering stage, *DFS* drought at flowering stage, *DGFS* drought at grain filling stage) of wheat.

Transpiration and photosynthesis rates were significantly dropped by 63.6, 57.8, 61.0, 44.2, 31.7, and 29.3% under drought stress at DTS, DFS, and DGFS, respectively, as compared to the control treatment. Moreover, stomatal conductance decreased positively under water stress by 37.8, 30.0, and 29.1% at DTS, DFS, and DGFS. Si-NPs treatment had a substantial effect on the gas exchange attributes of wheat leaves subjected to drought stress (Fig. 3A,B). Seed preparation of 300, 600, and 900 mg/L of Si-NPs significantly enhanced the transpiration and photosynthesis rate by 27.8, 34.6%,40.6 and 20.3, 26.8, 34.2%, respectively when compared with control. Seed preparation with 900 mg/L of Si-NPs application performed the best result than other treatments under drought.

### Water use efficiency and stomatal conductance

Si-NPs significantly affected the water use efficiency in wheat both under control and drought conditions, as indicated in Fig. 3C,D. WUE dropped by 43, 47, and 35% under drought stress at DTS, DFS, and DGFS, respectively, compared to the control treatment. Seed preparation of 300, 600, and 900 mg/L of Si-NPs significantly reduced the drought impact by 34%. 42% and 56%, respectively, as compared to no Si-NPs treatment. Similarly, stomatal conductance (m mol m^−2^ s^−1^) dropped by37.8, 30.0, and 29.2% under drought stress at DTS, DFS, and DGFS, respectively, compared to the control treatment. Moreover, seed preparation of 300, 600 and 900 mg/L of Si-NPs significantly improved stomatal conductance by 15.1, 31.6, and 41.6% as compared to control. In a drought condition, the highest results were achieved by seed priming with 900 mg/L of Si-NPs treatment.

### Effect of silicon nanoparticles and drought on enzymatic antioxidant

**Figure 4:**
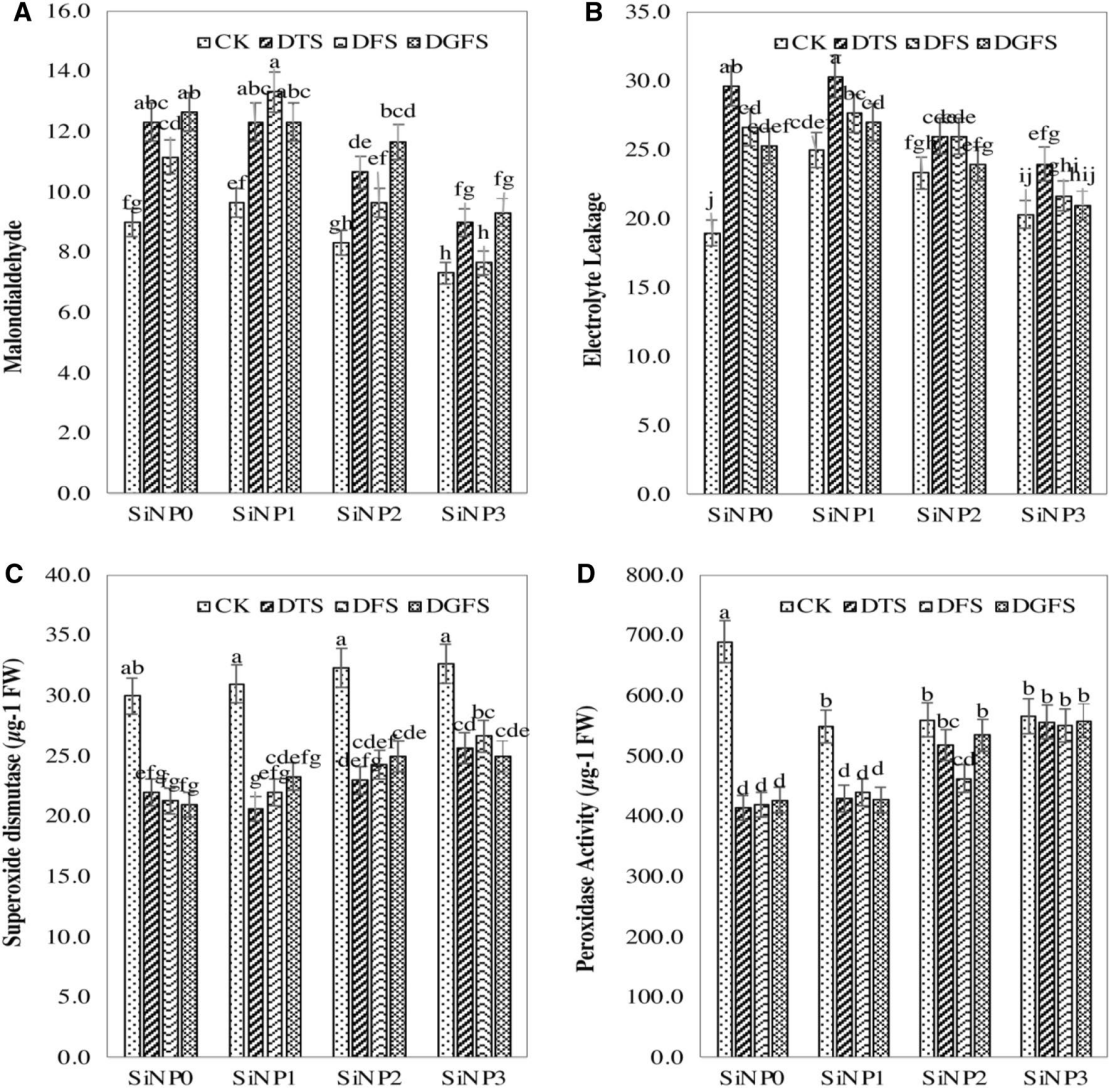
Effects of Si-NPs (0, 300, 600 and 900 mg/L) under drought stress on MDA **(A)**, EL% **(B),** SOD **(C)** and POD **(D)**. Critical growth stages of wheat were (*CK* Control, *DTS* drought at tillering stage, *DFS* Drought at the flowering stage, and *DGFS* Drought at the grain filling stage) of wheat.

To observe the oxidative injury in wheat leaves, the effect of Si-NPs was evaluated by estimating the concentration of ROS and antioxidant enzyme activities in Fig. 4. For this reason, the activities of SOD, POD, MDA, EL, and H_2_O_2_ were measured and higher amounts were seen in control plants. Moreover, at a higher dose of Si-NPs values of these above-mentioned parameters were reduced (Figs. 2D, 4A–D). The EL contents were diminished by 8.95 and 20%: MDA contents were diminished by 5.5, 16.2, and 31.7%; and the H_2_O_2_ were diminished by 11, 18, and 26% in seed preparing of 300, 600, and 900 mg/L of Si-NPs over the respective controls. The values of SOD and POD activities were measured in leaves to determine the activity of antioxidant enzymes. The treatments applied widely improved the ingredient of antioxidant enzymes, and the most extreme improvement was measured in the highest treatment of NPs. The SOD activity was expanded by 2, 10, and 16%, and POD action expanded by 5, 11, and 17% in 300, 600, and 900 mg/L NPs priming.

### Effect of silicon nanoparticles and drought on seed NPK %

**Figure 5:**
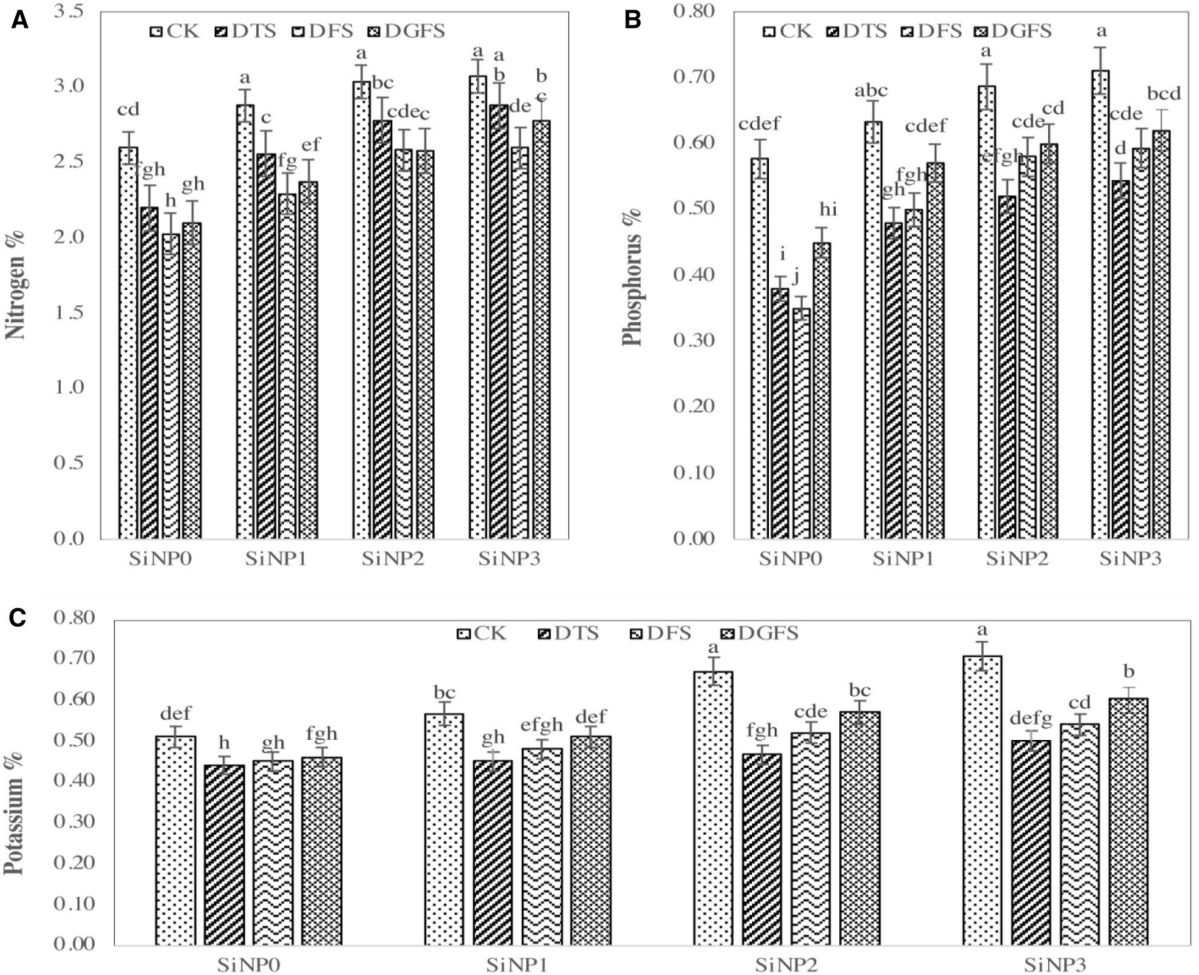
Effect of Si-NPs applications (0, 300, 600, and 900 mg/L) on seed (**A**) nitrogen percentage (N%), (**B**) phosphorus percentage (P%), and (**C**) Potassium percentage (K%) under drought stress at critical growth stages (*CK* control, *DTS* Drought at tillering stage, *DFS* drought at flowering stage, and *DGFS* drought at grain filling stage) of wheat.

The seed NPK values were significantly influenced by the application of Si-NPs under water-deficient conditions shown in Fig. 5. Drought stress decreased the wheat seed nitrogen% (11.2, 21.9 and 17.83%), P % (35.5, 28.8 and 16.3%), and K % (32.1, 23.2 and 14.3%) at different growth DTS, DFS and DGFS, respectively, when compared to control treatment. Silicon nanoparticle applications alleviated the adverse effects of water deficiency under control and stress environments. Seed preparing of 300, 600 and 900 mg/L of Si-NPs significantly enhanced the nitrogen% (13.1, 23.0 and 26.9%), P % (24.2, 35.8 and 40.4%), and K % (7.8, 19.7 and 26.4%), respectively when compared with control. The use of silicon nanoparticles enhanced the entire above-mentioned nutrient percentages, contrasted with the control treatment.

### Principal components analysis (PCA)

**Figure 6:**
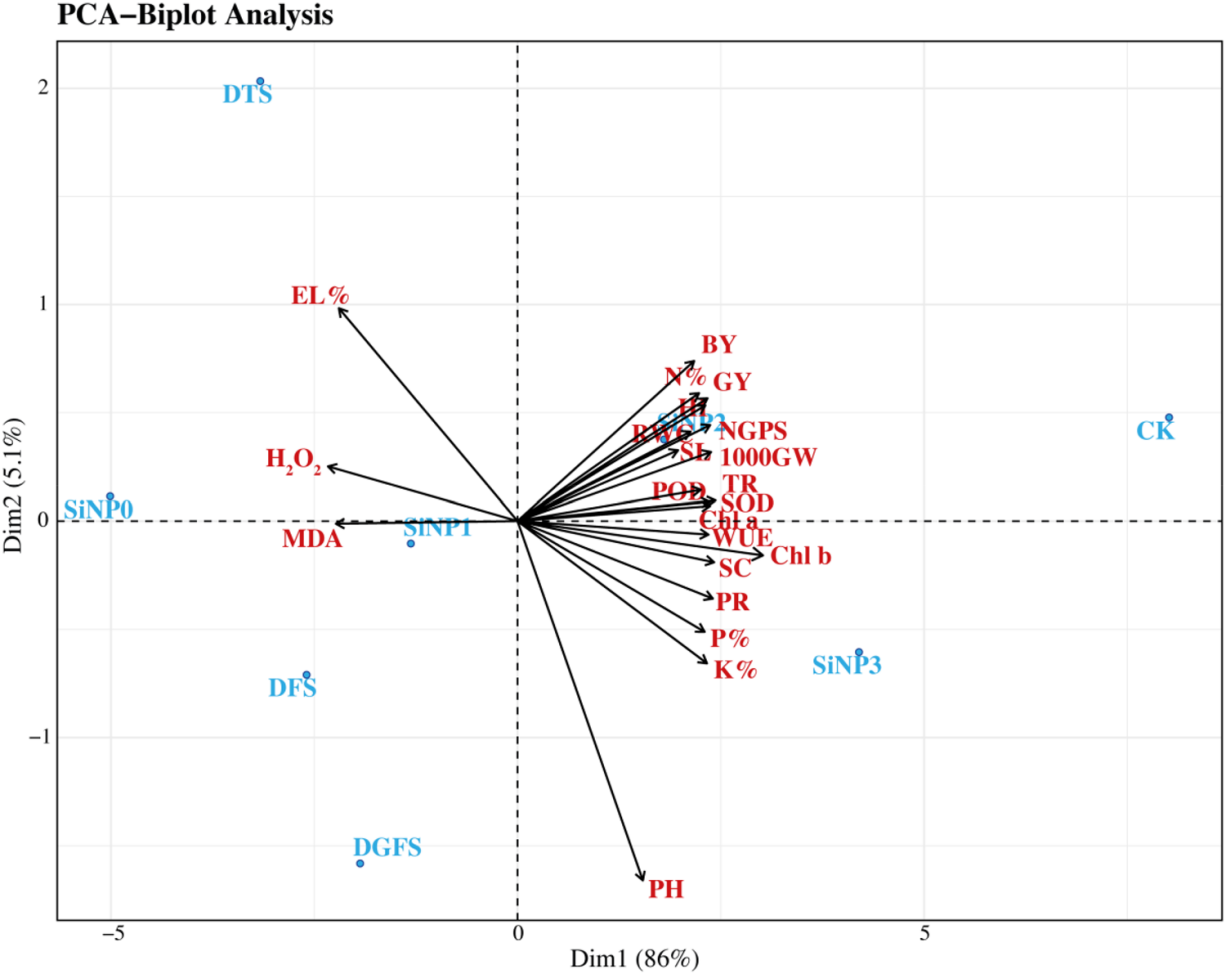
Principal component analysis (PCA) based on morpho-physiological and agronomic parameters of wheat under Si-NPs and drought stress. Plant height (PH, cm), spike length (SL, cm), number of grains per spike (NGPS), 1000-Grain Weight (GW, g), Grain Yield per Plant (GY, g), Biological Yield per Plant (BY, g), Harvesting Index (HI), Chlorophyll a (Chl a), Chlorophyll b (Chl b), Photosynthesis rate (PR), transpiration rate (Tr), Water use efficiency (WUE), stomatal conductance (SC), hydrogen peroxide (H_2_O_2_), malondialdehyde (MDA), superoxide dismutase (SOD), peroxidase (POD), and Electroleakage percentage (EL%). Application of Si-NPs (0, 300, 600 and 900 mg/L) under drought stress condition at different growth stages *CK* Control, *DTS* Drought at tillering stage, *DFS* drought at flowering stage, and *DGFS* drought at grain filling stage).

A principal component analysis (PCA) was performed to analyze the variations and associations among different morpho-physiological parameters of wheat under the application of Silicon nano-particles and drought stress. The treatments were scattered in different biplots, while the parameters were represented as vectors (Fig. 6). The first two PCs (PC1 86% and PC2 5.1%) covered the 91.1% variability of the total variation from the 22 parameters. The first PC was related to all the studied parameters except EL%, H_2_O_2_, and MDA which were attributed to PC2.All the treatments of Si-NPs (SiNP0 = 0, SiNP1 = 300, SiNP2 = 600, and SiNP3 = 900 mg/L) at different stages (CK = control, DTS = drought at tillering stage, DFS = flowering stage, and DGFS = grain filling stage) of wheat under drought stress showed variability among the treatments and growth stages. The amount of H_2_O_2_ was boosted under drought with negative eigenvalues (Fig. 6). Similarly, the combined effect of SiNPs and drought also reduced the MDA.

## Discussion

The primary objective of this study was to determine the optimal doses of Si-NPs for seed priming in order to accelerate wheat development and mitigate the negative effects of drought. Significant enhancements in growth characteristics were seen in seed primed with 900 mg/L under stress. Seed germination and plant growth are both negatively impacted by drought stress^47^. It has been observed by several studies that drought stress reduces the germination rate of wheat^23,35^. Drought has been shown to reduce seed germination, growth, and wheat yield^4,23,32^. Seed priming of nanoparticles enhanced the germination, seedling, roots structure, nutrient uptakes, and minimized the effect of water deficient conditions^18,20,37,41^.

Under drought, the plant height of wheat decreased significantly at different critical stages reported by Raza et al.^53^ Correspondingly, Aslam et al.^54^ observed that plant height was reduced in quinoa plants under water-limited conditions, which is parallel with our study. Liu and Lal^55^ stated that nano-particles are providing one more method to provide nutrients to plants under drought situations which is beneficial for plant height. Similarly, Paparella et al.^56^ found that seedlings become more vigorous at the early stages due to seed priming which prompts the metabolic system of wheat seed. Moreover, plant height was enhanced by the application of Si because it performed as a barrier when it was deposited in leaf apoplasts and protect the plant from stresses^57^. Silicon nano-particle seed priming beneficially enhanced the plant height of wheat under water-deficient conditions at critical stages of wheat growth. Because, Si may improve the protein substance, uptake of nutrients, photosynthetic rate and decrease the cell membrane in various plants^58,59^. Another plausible explanation is that plant height and morphological activities were enhanced due to the accumulation of water and nutrient in the seed during the time of priming with nanoparticles^60,61^. In this study, role of Si-NPs in wheat growth with and without drought was also studied.

Moreover, our outcomes exhibited photosynthesis activity enhanced positively with the application of Si-NPs (Fig. 2). As research revealed that chlorophyll contents directly affect plant growth and development. In our research, under Si-NPs application significantly enhanced the chl. a chl. b, carotenoid contents, and gas exchange parameters with and without stress conditions (Fig. 2). In the latest experiments, researchers observed a similar increment in photosynthesis with the application of nanoparticles^23,62,63^. The Si might reduce the drought stress influence on photosynthetic pigments by increasing cytokinin endogenously, which restorative the chlorophyll formation and improve chloroplast ultrastructure^64^. Besides this, Asgari et al.^36^ stated that it might be a result of NPs enhancing the nutrients in plants that improve photosynthesis. Furthermore, Si-NPs increased the thickness of the cell wall and enhanced the transport of nutrients by the opening of the xylem which is a major cause of enhancing the photosynthesis rate^36,65^.

Drought stress decreased photosynthesis rate, transpiration rate, stomatal conductance, water use efficiency, and relative water contents of leaves in plants under water-deficient conditions. There was a close connection between the photosynthesis, transpiration rate, and total chlorophyll focus for wheat growth under drought stress^66^. Abaaszadeh et al.^67^ concluded that the production of peroxidase, phenolic, and chlorophyllase was reduced under water stress conditions due to that chlorophyll concentrations were badly effect. Additionally, reducing water use efficiency and RWC in plants under drought stress diminished turgor pressure, water retention, and plant size. Comparative outcomes related to our study were noticed by Farooq et al*.*^68^, Zhao et al.^69^, and Mamnouie et al.^70^. Contrary to this, photosynthesis rate, transpiration rate, stomatal conductance, and RWC expanded as the result of Si-NPs application in both irrigation regimes, particularly in plants under drought stress. Zhu and Gong^71^ stated that these increments in gas exchange parameters may be due to Si potential to preserve the water under drought. Moreover, Si is accumulated under leaves cuticle, framing a twofold layer of Si-cuticle, consequently, the accumulation of Si has diminished the transpiration^72^. Accordingly, it is recommended that a silica-cuticle twofold layer framed on leaf epidermal tissue is responsible for higher RWC of leaves. In agreement with our results, Gong et al.^73^ found that the use of Na_2_SiO_3_ enhanced LA, dry mass, RWC, and leaf thickness of wheat plants under water stress. Likewise, Silica NPs improved water use efficiency, RWC, and chlorophyll content in maize crops^74,75^.

Si-NPs application significantly enhanced the action of SOD and POD while, H_2_O_2_, EL, and MDA substances were decreased significantly shown in Fig. 2D and Fig. 4A–D. Furthermore, the significant decrease in reactive oxygen species was due to the maintenance and recovery of the cell membrane in wheat plants with the application of Si-NPs^76,77^. Similarly, many researchers reported that nano-particles rise the activity of antioxidants in many crops^23,62,78,79^. Consequently, the application of silicon enhances the activities of SOD, glutathione reductase^80^, and catalase^81^ moderately balancing the adverse effect of drought on plants.

The absorption of nutrients including N, P, and K by plants is also negatively impacted by drought stress. It has been shown in a number of studies that drought stress causes a decrease in both the content of N, K, and P in plant tissue and the rate of nutrient absorption from soil. It is well-documented that soybean N content drops significantly during drought stress^78^. A significant loss of nitrogen and phosphorus in plant tissues was documented by Rizwan et al.^23^ under situations of drought stress. The current research found that, compared to the control, Si-NPs significantly enhanced N, P, and K content under drought circumstances.

In drought conditions, wheat growth and development are diminished due to fluctuating in the balance of nutrients^82^. Wheat yield and biomass are enhanced by enhancing the number of Si-NPs (0–900 mg/L) shown in Table 2. The seed priming of Si-NPs demonstrates the advantageous impacts on wheat plants. As previous research reviled that molecular, biochemical, and physiological changes due to drought stress have negatively impacted plant growth and yield^83^. Such as dry matter production in plants decreases due to the limitation of photosynthesis and stomatal closure which reduced the carbon dioxide concentration under drought stress^84^. Moreover, Yazdanpanah et al.^85^ stated that under drought stress excessive accumulation of ROS which causing the oxidative damage of lipids, proteins, and DNA in plants which decreased plant growth and development. Similarly, other researchers reported that drought stress decreases the biomass, grain, and yield of wheat^86,87^.

Furthermore, the results exhibited that utilization of some concentrations of Si-NPs increased biomass, plant height, yield, and yield components in both irrigation regimes. Generally, the positive effect of Si application in plants is not too obvious under optimum conditions, but it is most evident when the plant is under suboptimal conditions^88^. However, Si-NPs improved the destructive effect of drought on the growth, yield, and its components due to a variation in transpiration, improvement in photosynthesis rate, and plant water status^33,89^. These findings are in line with Sharifi Rad et al.^90^ and Shallan et al.^91^. Si-NPs have the potential to boost the RWC, chlorophyll contents, ROS activities, growing conditions, and yield of crops^34,92,93^.

## Conclusions

The results exposed that the Silicon nanoparticles (Si-NPs) seed priming under drought conditions significantly enhances the growth of wheat and reduces water stress. The biomass, plant height, and yield of the wheat crop were boosted due to Si-NPs seed priming. It can be concluded that enhancing the concentration of Si-NPs in wheat plants significantly enhanced the number of antioxidant enzymes, chlorophyll contents, and gas exchange parameters, and minimized the water stress. Moreover, seed priming Si-NPs enhanced the enzyme activities that minimized the concentrations of ROS in wheat leaves. Therefore, the results suggest that seed priming application of Si-NPs can be helpful to wheat plants either in normal irrigation or in drought stress. Though, further investigations at pot and field levels are still needed to see how nano-particles initiate this impact on drought stress in cereals by the priming application of NPs under drought conditions.

## Data Availability

All data generated or analysed during this study are included in this published article.
